# Protective potentials of polymyxin B and honey against bacterial lipopolysaccharide-induced endotoxemia in mice

**DOI:** 10.5455/javar.2024.k800

**Published:** 2024-06-22

**Authors:** Ferdous Hasan Mithun, Md. Eftakhar Jahan Bhuiyan, Md. Golzar Hossain, Chirojit Debnath, K. H. M. Nazmul Hussain Nazir, Sharmin Akter

**Affiliations:** 1 Department of Physiology, Bangladesh Agricultural University, Mymensingh, Bangladesh; 2 Department of Microbiology and Hygiene, Bangladesh Agricultural University, Mymensingh, Bangladesh; 3 Department of Hepatology, Mymensingh Medical College, Mymensingh, Bangladesh

**Keywords:** Hemato-biochemistry, histopathology, honey, LPS, polymyxin B, survival rate

## Abstract

**Objective:**

The experiment aimed to determine the effects of lipopolysaccharide (LPS), polymyxin B, and honey on survival rates, hematological parameters, liver and kidney biomarkers, blood glucose levels, serum insulin levels, and histopathology of the liver, kidney, lungs, brain, and pancreas in LPS-challenged mice.

**Materials and Methods:**

50 male Swiss Albino mice (*Mus musculus*), aged 3 weeks, were randomly assigned into 5 groups (10 mice per group): Control group (A), LPS (2 mg/kg bwt/day IP in NS) treated group (B), polymyxin B (1.2 mg/kg bwt/day IM) pre-treated plus LPS (2 mg/kg bwt/day IP in NS) treated group (C), honey (10 gm/kg bwt/day PO) pre-treated plus LPS (2 mg/kg bwt/day IP in NS) treated group (D), both polymyxin B (1.2 mg/kg bwt/day IM) and honey (10 gm/kg bwt/day PO) pre-treated plus LPS (2 mg/kg bwt/day IP in NS) treated group (E). The LPS was administered intraperitoneally (IP) at 80 µg/mice/day, diluting in normal saline. After 16 weeks, the mice were sacrificed, and blood samples and organs (liver, kidney, lung, brain, and pancreas) were collected for laboratory tests.

**Results:**

The results revealed that in LPS-treated mice, the mortality rate was the highest, and hemato-biochemical parameters were altered. Histopathological examination in the group treated with LPS showed disarrangement of hepatocytes, cellular infiltrations in the glomerulus, alveolar congestion in the lungs, several nerve fiber degenerations in the brain, and degenerative changes in pancreatic islets. The mortality rate and hemato-biochemical and histopathological changes were restored by the combined treatment of polymyxin B and honey.

**Conclusion:**

LPS has detrimental effects on survival rate and hemato-biochemistry, which are lessened by taking honey and polymyxin B supplements.

## Introduction

According to recent views, sepsis is a potentially fatal organ failure that results from an uncontrolled body reaction to an infection. Metabolic, cellular, and hemodynamic changes significantly raise the risk of death in septic shock, a type of sepsis [1]. According to the World Health Organization (WHO), more than thirty million individuals experience septic complications each year, and 6 million of them pass away globally. Antimicrobial stewardship is compromised by the global increase in multidrug-resistant bacterial sepsis, especially caused by Gram-negative bacteria, which also raises mortality and morbidity rates [2]. Especially, *Escherichia coli *(*E. coli*), the opportunistic niche pathogens, that are ubiquitous in the environment and largely affect seriously ill and immunocompromised individuals, as well as small infants, especially neonates, and young newborns, particularly neonates, and are notorious for developing multiresistance to available antibiotics. Endotoxemia from Gram-negative infections appears to be highly prevalent in seriously sick and injured patients.

Endotoxins, also called lipopolysaccharides (LPS), are macromolecular glycolipids in Gram-negative bacteria’s outer membrane, released during growth, division, and lysis [3]. LPS refers to purified LPS, while endotoxin is LPS attached to a bacterial membrane, isolated from Enterobacteriaceae, Pseudomonadaceae, Pasteurellaceae, and Vibrionaceae. *Escherichia coli* is transmitted through contaminated food and water via the oro-fecal route, affecting both humans and animals. However, it regulates gut motility, synthesizes vitamins, ferments food particles, and influences microglia development in the central nervous system, but its massive colonization may become detrimental to human and animal health. Gut bacteria *E. coli* and their LPS do not harm gut epithelial cells, but toxicity occurs when LPS reaches the basal side, causing bacteria to penetrate mucus, damaged enterocytes, and deeper tissues, and then it produces excessive quantities of LPS. On the other hand, increased gastrointestinal permeability and the consequent transfer of germs or bacterial metabolites can cause endotoxin levels in the blood to spike 1,000-fold in sepsis [4]. LPS are universal inflammatory agents affecting various systems, including high fever, intravascular coagulation, multiorgan failure, liver dysfunction, kidney failure, septic shock, and even the death of animals [5].

Polymyxin B is a lipopeptide antibiotic from *Bacillus polymyxa* with detergent-like properties and dual action, killing bacteria and disrupting LPS. Polymyxins, classified as A-E, have been used in human and veterinary medicine for over 50 years. Currently, only B and E are used for treating Gram-negative bacterial infections. Polymyxin B’s pharmacokinetics and pharmacodynamics were limited before the 1980s, but usage has increased significantly in recent decades. Polymyxin B, with a high affinity for endotoxin, can cause nephrotoxicity and neurotoxicity in humans and animals, destroying membrane integrity. As a result, their prolonged usage was suspended until the mid-1990s [6]. Polymyxin B hemoperfusion therapy, available in Japan for over a decade, effectively binds endotoxin, treating thousands of patients. However, no large randomized trials have established its efficacy. However, perceived nephrotoxicity and neurotoxicity have been a significant hindrance to their early and frequent administration in severe sepsis cases. Recent studies suggest polymyxins may be resistant to bacteria by altering membrane structure and restricting antibiotic interaction, which is our last hope for antibiotics [6]. Polymyxin resistance, particularly hetero-resistance, is a growing concern, and antibiotics can disrupt gut microbiota, altering immune systems. Honey, a natural antioxidant, may be used to treat endotoxemia. It is a natural, sugared, viscous liquid produced from nectar by bees that includes approximately 400 substances such as proteins, enzymes, organic acids, vitamins, flavonoids, and volatiles. Historically, honey has treated wounds, burns, and ulcers by stimulating the immune system, boosting white blood cells, countering inflammation, clearing infections, and promoting cell growth [7]. Honey has been described as ameliorating metabolic dysfunction, being capable of increasing hematological parameters [hemoglobin (Hb) content, red blood cell (RBC), PCV, platelet, and white blood cell (WBC)], decreasing oxidative stress, reducing the inflammatory response in mice, and balancing the blood glucose level and final body weight [8]. Therefore, honey is the primary focus of this study.

Though many studies have found that the LPS of *E. coli O55:B5 *induces septic shock, there is a very limited investigation of polymyxin B sulfate and honey in such sepsis. Hence, this study aims to assess the potency of the antibiotic polymyxin B and the natural antibacterial substance, honey, on physiological performance and organ functions in endotoxemic mice.

## Materials and Methods

### Ethical statement

All the procedures and protocols applied in this animal experiment were approved by the Animal Welfare and Experimentation Ethics Committee (AWEEC/BAU/2022(63)) at Bangladesh Agricultural University (BAU).

### Materials

L2880-10MG (Lot # 0000114327) of LPS (*Escherichia coli* O55:B5) was bought from the Sigma-Aldrich Company, Spruce Street, St. Louis, USA. Injection Polymax B of 5,00,000 units was procured from Incepta Pharmaceuticals Ltd. Natural bee honey from the Sundarbans in Bangladesh was used for the experimentation. Physical and biochemical tests of honey were performed using standard protocols [9].

### Animals and treatments

The mice reared for this experiment were bought from the International Center for Diarrheal Disease Research, Bangladesh, Mohakhali, Dhaka. Fifty male Swiss Albino mice (*Mus musculus*), aged at 3 weeks, were arbitrarily divided into 5 groups (*n =* 10); control (A), LPS (2 mg/kg bwt/day IP in NS) [10], treated group (B), and polymyxin B (1.2 mg/kg bwt/day IM) [11] pre-treated plus LPS (2 mg/kg bwt/day IP in NS) treated group (C), honey (10 gm/kg bwt/day PO) [12] pre-treated plus LPS (2 mg/kg bwt/day IP in NS) treated group (D), both polymyxin B (1.2 mg/kg bwt/day IM) and honey (10 gm/kg bwt/day PO) pre-treated plus LPS (2 mg/kg bwt/day IP in NS) treated group (E). A mouse model of endotoxemia was performed intraperitoneally (IP) and injected with LPS (*Escherichia coli* O55:B5, Difco Laboratories, Detroit, MI) at 80 µg/mice/day diluting in NS. The experiment was carried out for 16 weeks.

### Chemical administration and survival rate observation

Pure Bee honey was purchased from the market, and it was administered orally at 10 gm/kg bwt/day after mixing with a mouse pellet for 32 days. Polymyxin B sulfate was administered by intramuscular injection at 1.2 mg/kg bwt for 5 consecutive days before the LPS treatment. LPS was administered through intraperitoneal injection at 80 µg/mice/day after diluting with normal saline. The survival rate of mice in each group was checked at 24-h intervals of LPS injection up to 72 h.

### Sample collection and processing

Blood samples were collected after 72 h of LPS injection by sacrificing the mice. About 1.5 ml of blood was collected from the hearts of mice by a sterile syringe under anesthetic conditions using diethyl ether (Fine Chem Industries, India) anesthesia. 1 ml of the collected blood sample was kept in an anticoagulant tri-sodium (Fine Chem Industries, India) containing an Eppendorf tube for hematological tests, while the remaining half was kept in another tube without anticoagulant for preparing serum. Then, the serum was separated by centrifuging the blood at 1,500 rotations per minute for 30 min [13]. The serum samples were kept at −20°C for biochemical studies. After 72 h of LPS injection, mice were sacrificed, and tissues (lung, liver, kidney, intestine, brain, and pancreas) were collected from the sacrificed mice for observing histological changes. The tissue samples were preserved in 10% neutral buffer formalin for 24 h for further processing [14].

### Hematological examination

Collected blood samples were analyzed to determine the total erythrocyte count (TEC), concentration Hb, packed cell volume (PCV), total leukocyte count (TLC), and differential leukocyte count (DLC) according to the standard protocols [15].

### Biochemical examination

Blood samples from the experimental mice were examined for aspartate aminotransferase (AST) and alanine transaminase (ALT), serum urea and creatinine levels, serum glucose, and insulin levels using a Reflotron^®;^ auto-analyzer (Boehinger Mannheim, Germany) with a particular test kit. The laboratory experiments were carried out at Bangladesh Agricultural University’s Professor Mohammad Hussain Central Laboratory in Mymensingh.

### Observation of histopathological changes

After the collection of blood samples, liver, lung, heart, kidney, brain, and pancreatic tissues were collected and stored in 10% neutral buffered formalin for 24 h. The specimens were fixed, sectioned, and rinsed overnight in running tap water. Afterward, the tissues were dehydrated in an escalating series of 50%, 70%, 80%, 90%, and 100% ethanol for 1 h. Then the tissues were washed in chloroform for 3 h (one and a half hours each, twice) [14]. The specimen was embedded in molten paraffin (56°C–60°C) for 3 h. The tissues were then sectioned using a microtome, and then a small quantity of gelatin was added to the water bath to improve the adhesiveness of the slide section. The sections were allowed to spread in a heated water bath set to 40°C–42°C. The sections were taken on grease-free, transparent slides. Finally, the slides with the sections were air-dried and stored in a cold area. The stained slides were assessed using the OLYMPUS CX41 microscope at 10X and 40X magnification.

### Analysis of the statistical data

All experimental data were statistically analyzed with GraphPad Prism (9.3.1). Data were presented as mean ± SD. Two mouse groups were compared using ANOVA followed by Tukey’s multiple comparison tests, with statistical significance set at *p* < 0.05.

## Results

### Effect of LPS, polymyxin B, and honey on the survival rate of mice

**Figure 1.**
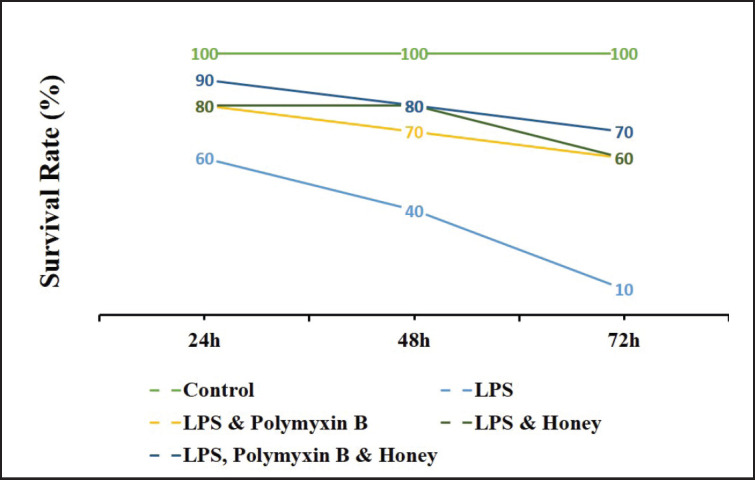
Effect of LPS, polymyxin B, and honey on survival rate in mice. The values of the serum survival rate of mice of different treatment groups are presented in the graph. The groups were untreated control mice, LPS-treated mice, LPS plus polymyxin B-treated mice, LPS plus honey-treated mice, and LPS plus polymyxin B plus honey-treated mice. The survival rate was checked 24 h of LPS injection interval at a rate that ended at 72 h.

The survival rate of mice in different treatment groups is presented in Figure 1. Results revealed that the survival rate of mice in LPS-treated mice was the lowest among the groups. Whereas, it was higher in the polymyxin B and honey-treated groups and highest in the untreated control group.

### Effect of LPS, polymyxin B, and honey on red blood cell (RBC) parameters

**Figure 2.**
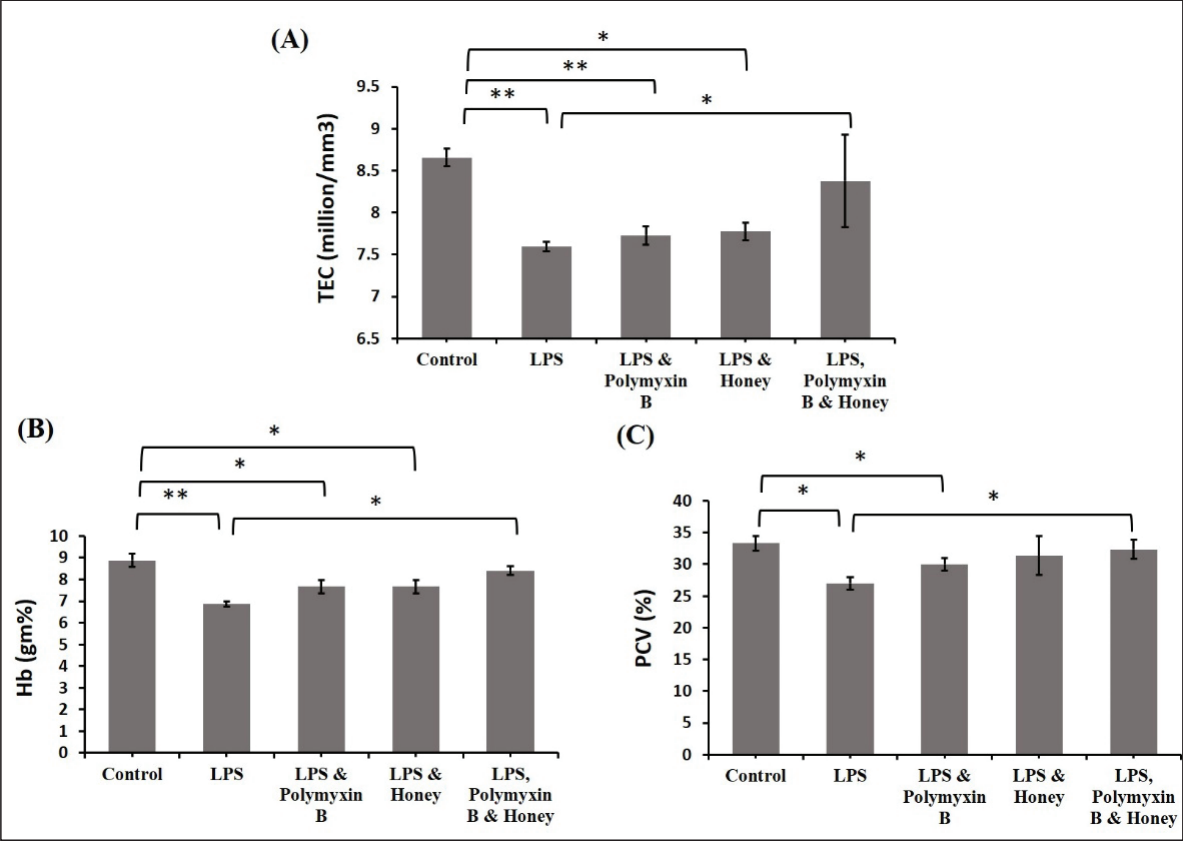
Effect of LPS, polymyxin B, and honey on TEC, Hb concentration, and PCV in mice. The mean values of TEC, Hb concentration, and PCV of mice of different treatment groups are presented in the graph. Untreated control mice, LPS-treated mice, LPS plus polymyxin B treated mice, LPS plus honey-treated mice, and LPS plus polymyxin B plus honey-treated mice. Data are shown as mean ± SD. The differences between the two groups of animals were compared by using one-way repeated measure ANOVA followed by Tukey’s multiple comparisons tests. Here, **= Statistically significant at 1% level (*p < *0.01), *= Statistically significant at 5% level (*p < *0.05).

The effects of LPS, polymyxin B, and honey on TEC, Hb, and PCV in different mouse groups were examined (Fig. 2). LPS-treated mice had significantly lower TEC (*p < *0.01) compared to untreated controls. In mice treated with LPS and polymyxin B or LPS and honey, TEC declined significantly (*p < *0.01) and (*p < *0.05) compared to untreated control mice (Fig. 18). Mice treated with LPS, polymyxin B, and honey showed significantly higher TEC values (*p < *0.05) than mice treated with only LPS. In LPS-treated mice, Hb concentration and PCV were considerably (*p < *0.01) lower than in untreated controls. Mice treated with LPS and polymyxin B or LPS and honey showed a modest rise in Hb concentration and PCV compared to the just LPS-treated mice. However, the mice treated with LPS, polymyxin B, and honey showed a substantial increase (*p < *0.05).

### Effect of LPS, polymyxin B, and honey on WBC parameters

**Figure 3.**
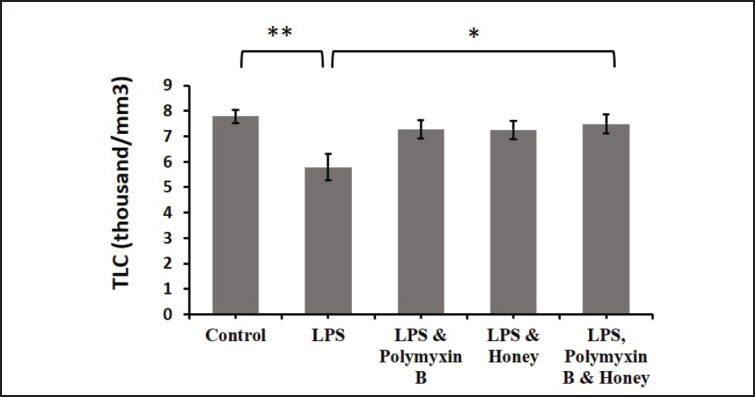
Effect of LPS, polymyxin B, and honey on TLC in mice. The mean values of TLC of mice of different treatment groups are presented in the graph. Untreated control mice, LPS-treated mice, LPS plus polymyxin B treated mice, LPS plus honey-treated mice, and LPS plus polymyxin B plus honey-treated mice. Data are shown as mean ± SD. The differences between the two groups of animals were compared by using one-way repeated measure ANOVA followed by Tukey’s multiple comparisons tests. Here, **=Statistically significant at 1% level (*p *< 0.01), *= Statistically significant at 5% level (*p *< 0.05).

The impacts of LPS, polymyxin B, and honey on TLC in different groups of mice were analyzed (Fig. 3). The results revealed that the TLC in the LPS-treated group was significantly (*p *< 0.01) reduced compared to the untreated control mice. Similarly, TLC in the mice treated with either LPS and polymyxin B or LPS and honey also showed a slight declination compared to the untreated control group. However, TLC in mice groups either treated with LPS and polymyxin B or LPS and honey showed a slight increase compared to the only LPS-treated mice, but significantly (*p *< 0.05) increased in mice groups treated with LPS, polymyxin B, and honey.

**Figure 4.**
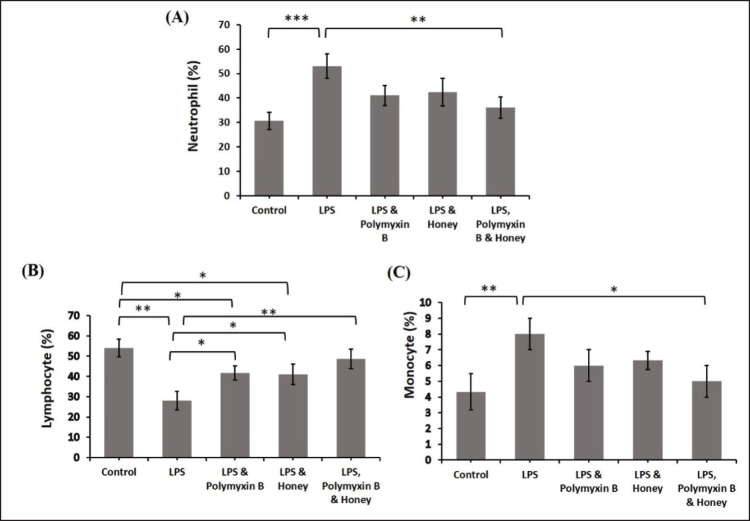
Effect of LPS, Polymyxin B, and Honey on DLC in mice. The mean values of neutrophil, lymphocyte, and monocyte of mice of different treatment groups are presented in the graph. Group A-Control, Group B-LPS treated group, Group C-LPS and Polymyxin B treated group, Group D-LPS and Honey treated group, Group E-LPS, Polymyxin B and Honey treated group. Data are shown as mean ± SD. The differences between the two groups of animals were compared by using one-way repeated measure ANOVA followed by Tukey’s multiple comparisons test. Here, ***= Statistically significant at 0.1% level (*p *< 0.001), **= Statistically significant at 0.1% level (*p *< 0.01), *= Statistically significant at 5% level (*p *< 0.05).

Analyses were conducted on the effects of LPS, polymyxin B, and honey on DLC in several mouse groups (Fig. 4). When comparing the LPS-treated mice to the untreated control mice, there was a significant (*p < *0.001) increase in the percentage of neutrophils and monocytes. Comparing the treated mice group to the untreated control mice, the percentage of mice treated with LPS and polymyxin B or LPS and honey also increased (*p < *0.05). On the other hand, compared to mice treated with just LPS, mice treated with polymyxin B and honey showed considerably lower percentages of neutrophils and monocytes (*p < *0.01) and (*p < *0.05). Regarding lymphocytes, there was a statistically significant (*p < *0.01) decrease in the proportion between the LPS-treated group and the untreated control mice. Similarly, lymphocytes in the mice group treated with LPS and polymyxin B or LPS and honey were significantly reduced (*p < *0.05) compared to untreated control mice. The percentage of mice treated with LPS and polymyxin B or LPS and honey improved significantly (*p < *0.05) compared to group B, as did the percentage of mice treated with LPS, polymyxin B, and honey (*p < *0.01).

### Effect of LPS, polymyxin B, and honey on biochemical parameters

The effects of LPS, polymyxin B, and honey on biochemical parameters such as AST, ALT, urea, creatinine, glucose, and insulin were studied in the experiment to understand the function of the liver, kidney, and other body systems.

### Effect of LPS, polymyxin B, and honey on liver function tests

**Figure 5.**
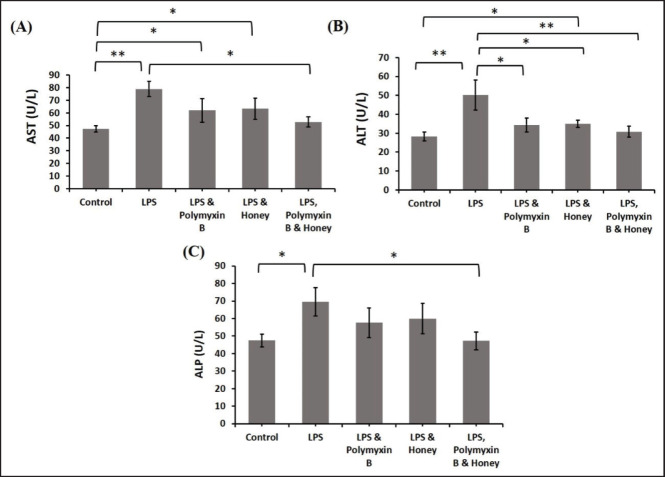
The impact of LPS, polymyxin B, and honey on liver function test in mice. The mean values of AST, ALT, and ALP of mice of different treatment groups are presented in the graph. Untreated control mice, LPS-treated mice, LPS plus polymyxin B treated mice, LPS plus honey-treated mice, and LPS plus polymyxin B plus honey-treated mice.Data are shown as mean ± SD. The differences between the two groups of animals were compared by using one-way repeated measure ANOVA followed by Tukey’s multiple comparisons tests. Here, **= Statistically significant at 1% level (*p *< 0.01), and *= Statistically significant at 5% level (*p *< 0.05).

The effects of LPS, polymyxin B, and honey on serum AST, ALT, and alkaline phosphatase (ALP) levels in different mouse groups were examined (Fig. 5). LPS treatment significantly increased AST and ALT levels (*p < *0.01) compared to group A. The mice treated with LPS and polymyxin B or LPS and honey had significantly higher (*p < *0.05) AST and ALT levels compared to the untreated controls. Mice treated with LPS, polymyxin B, and honey showed significantly lower levels of AST and ALT (*p < *0.05) compared to those treated with only LPS. LPS-treated mice had significantly higher levels of ALP (*p < *0.05) than untreated control mice, similar to AST and ALT levels. Whereas, the LPS, polymyxin B, and honey-treated mice showed significantly lower ALP levels (*p < *0.05) compared to LPS-treated mice alone.

### Effect of LPS, polymyxin B, and honey on kidney function tests

**Figure 6.**
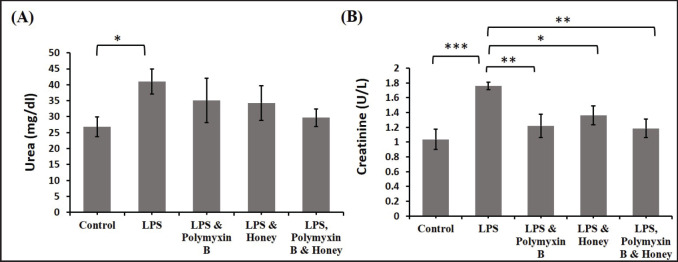
Effect of LPS, polymyxin B, and honey on kidney function test in mice. The mean values of urea and creatinine levels of mice of different treatment groups are presented in the graph. Untreated control mice, LPS-treated mice, LPS plus polymyxin B treated mice, LPS plus honey-treated mice, and LPS plus polymyxin B plus honey-treated mice. Data are shown as mean ± SD. The differences between the two groups of animals were compared by using one-way repeated measure ANOVA followed by Tukey’s multiple comparisons tests. Here, **= Statistically significant at 1% level (*p *< 0.01), *= Statistically significant at 5% level (*p *< 0.05).

The effects of LPS, polymyxin B, and honey on urea and creatinine levels in various mouse groups were investigated (Fig. 6). LPS-treated mice had significantly higher serum urea levels (*p < *0.05) compared to the control group. In contrast, the urea level in mice treated with LPS and polymyxin B or LPS and honey was slightly lower than in mice treated with only LPS, but it was even lower in mice treated with LPS, polymyxin B, and honey. Similar to urea, LPS-treated mice had significantly higher creatinine levels (*p < *0.001) than untreated control mice. Similarly, creatinine levels were higher in the mice treated with either LPS and polymyxin B or LPS and honey than in the untreated control mice. Conversely, mice in group D showed a significant (*p *< 0.05) reduction compared to group B. Similarly, the creatinine level was also significantly (*p *< 0.01) reduced in the mice group either treated with LPS and polymyxin B or LPS, polymyxin B, and honey.

**Figure 7.**
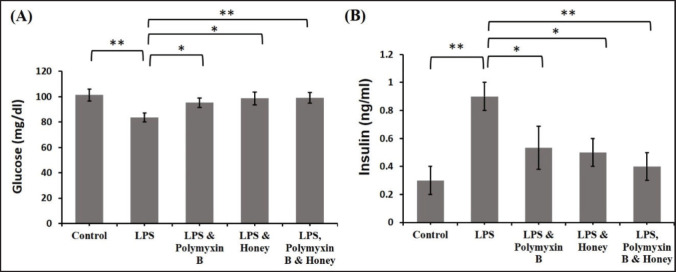
Effect of LPS, polymyxin B, and honey on serum glucose and insulin level in mice. The mean values of the serum glucose and insulin level of mice of different treatment groups are presented in the graph. Untreated control mice, LPS-treated mice, LPS plus polymyxin B treated mice, LPS plus honey-treated mice, and LPS plus polymyxin B plus honey-treated mice*. *Data are shown as mean ± SD. The differences between the two groups of animals were compared by using one-way repeated measure ANOVA followed by Tukey’s multiple comparisons tests. Here, **= Statistically significant at 1% level (*p *< 0.01), *= Statistically significant at 5% level (*p *< 0.05).

### Effect of LPS, polymyxin B, and honey on serum glucose and insulin levels

The effects of LPS, polymyxin B, and honey on serum glucose and insulin levels in different groups of mice were analyzed (Fig. 7). Results revealed that the serum glucose levels in LPS-treated mice were significantly (*p *< 0.01) decreased compared to the untreated control mice. Similarly, it decreased slightly in treated mice with either LPS and polymyxin B or LPS and honey compared to mice in group A. Whereas the serum glucose level in mice groups either treated with LPS and polymyxin B or LPS and honey was elevated significantly (*p *< 0.05) compared to group B as well as significantly (*p *< 0.01) increased in mice groups treated with LPS, polymyxin B, and honey.

LPS-treated mice showed significantly lower serum insulin levels (*p < *0.01) than untreated control mice. Similarly, it increased in mice treated with LPS, polymyxin B, or honey. Serum insulin levels in mice treated with LPS and polymyxin B or LPS and honey decreased slightly (*p < *0.05) compared to LPS-treated mice alone, but significantly (*p < *0.01) in the group treated with LPS, polymyxin B, and honey.

### Effect of LPS, polymyxin B, and honey on the histopathology of the liver, kidney, lung, brain, and pancreas

**Figure 8.**
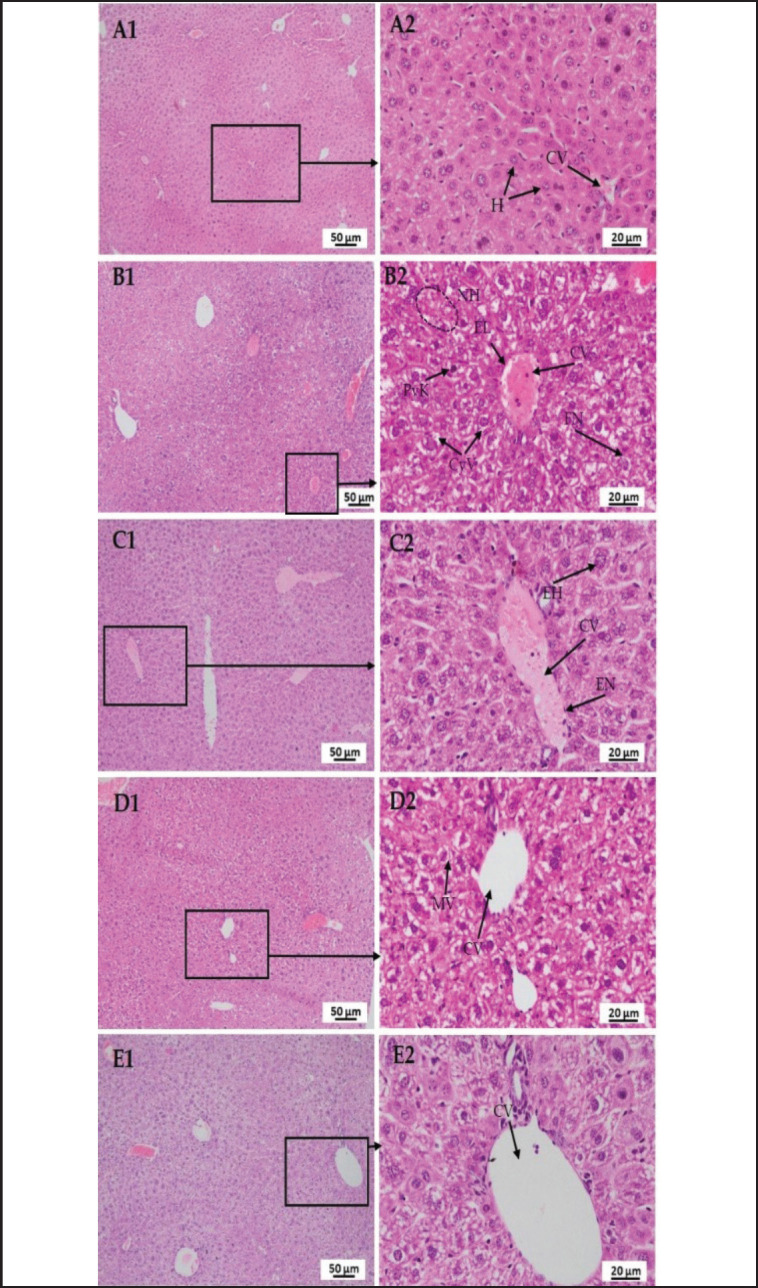
Photomicrograph of histo-structures of liver of different treatment groups.Group A-Control, Group B-LPS treated group, Group C-LPS and Polymyxin B treated group, Group D-LPS and Honey treated group, Group E-LPS, Polymyxin B and honey treated group. Control group A showed normal tissue structures with a regularly organized hepatic cord, and central vein (CV) with normal hepatocytes (NH).But in the case of LPS-treated group B, disarrangement of hepatocytes along with pyknosis (Pyk), cytoplasmic vacuoles (CyV), fragmented nucleus (FN), and inflammatory cells and congestion in the central vein (CV). In the case of group C, intact lining endothelial cells (EN denotes endothelial nucleus) around the central vein, mild congestion in the central vein (CV), and a smaller number of enlarged hepatocytes (EH). In the case of group D, mild vacuoles (MV) and very minor cellular degeneration were observed. However, in group E, there were no major detectable changes in the hepatic cord and central vein.

**Figure 9.**
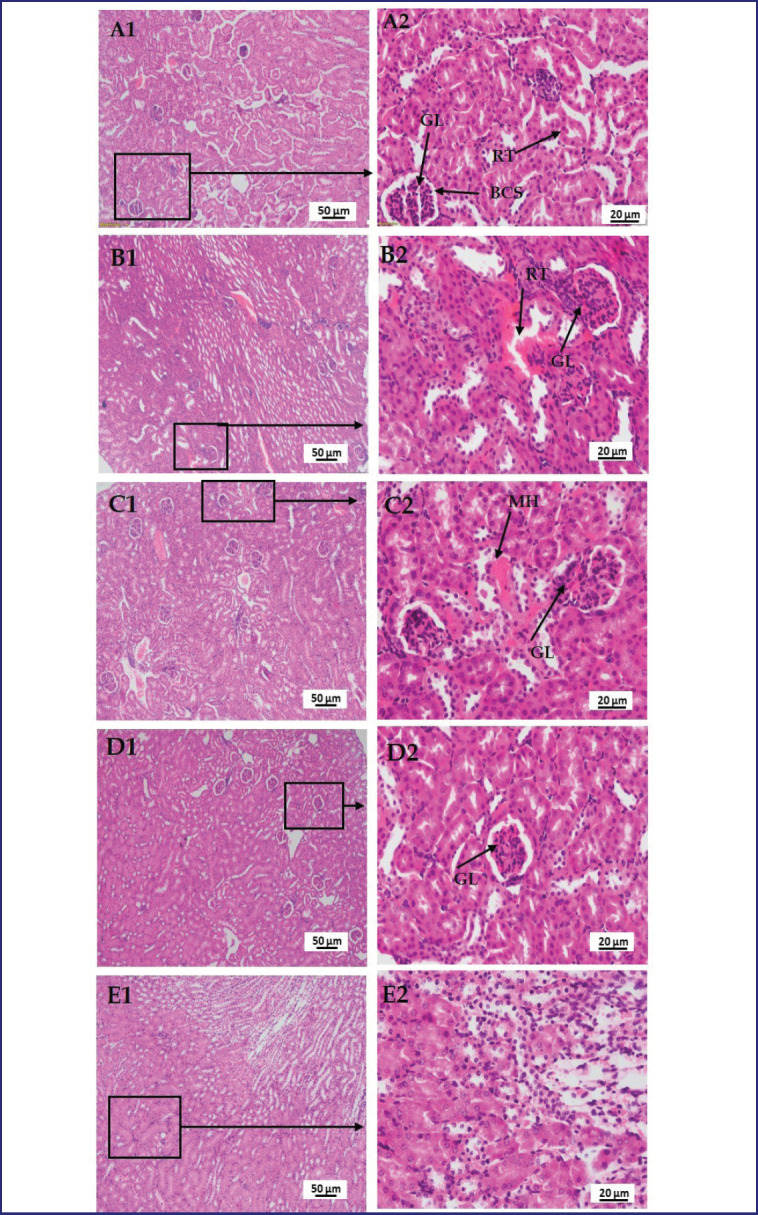
Photomicrograph of histo-structures of kidney of different treatment groups.Group A-Control, Group B-LPS treated group, Group C-LPS and Polymyxin B treated group, Group D-LPS and Honey treated group, Group E-LPS, Polymyxin B and honey treated group. Control group A showed normal tissue structures with regularly organized glomerulus (GL), bowman’s capsular space (BS), and convoluted renal tubules (RT). But in the case of LPS-treated group B, numerous cellular infiltrations in the glomerulus (GL) and hemorrhage in renal tubules (RT). In the case of group C, mild congestion and relatively less glomerular infiltration. In the case of group D, no hemorrhage and no significant glomerular infiltration. However, in group E, there were no detectable changes rather than a smaller number of cellular infiltrations.

**Figure 10.**
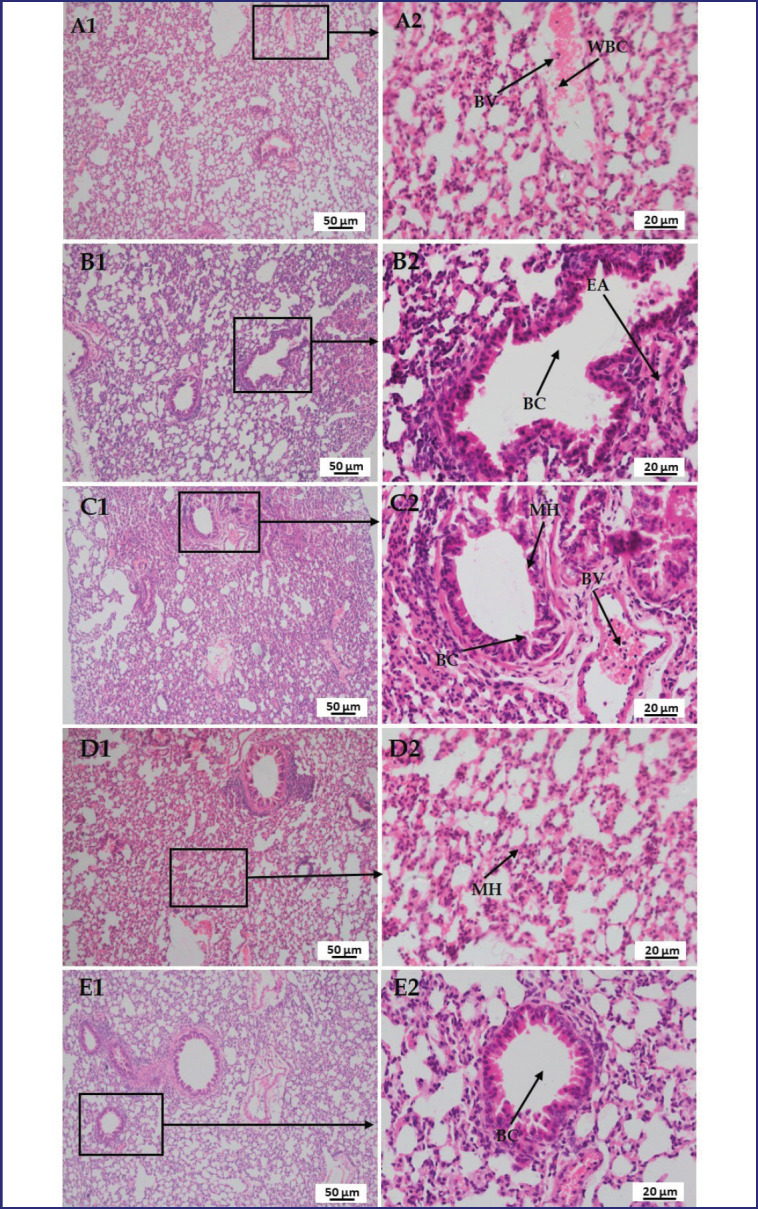
Photomicrograph of histo-structures of lung of different treatment groups.Group A-Control, Group B-LPS treated group, Group C-LPS and Polymyxin B treated group, Group D-LPS and honey treated group, Group E-LPS, Polymyxin B and honey treated group. Control group A showed normal structures of blood vessel (BV) rather than cuboidal shape pneumocytes. Whereas, in the case of LPS-treated group B, distended or ruptured bronchiole (BC) along with peri-bronchiole cellular infiltration and misshaped or emphysematous alveoli (EA) were observed. In the case of group C, mild hemorrhage (MH) and peri-bronchial cell infiltration (BC), and normal venioles (BV) with the ratio of RBCs and WBCs. In the case of group D, showed minor hemorrhage (MH) in the alveoli. However, in group E, there were no detectable rather than very a smaller number of cellular infiltrations around the bronchiole (BC).

**Figure 11.**
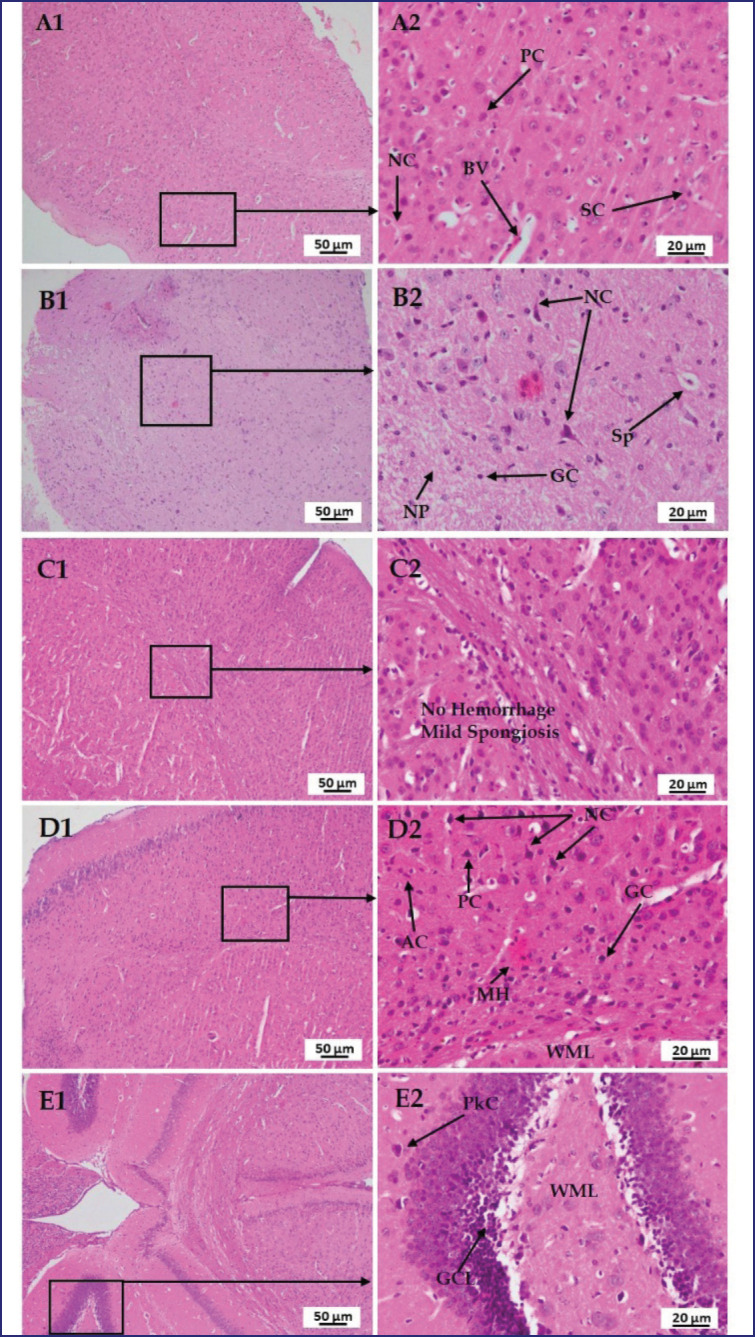
Photomicrograph of histo-structures of brain of different treatment groups.Group A-Control, Group B-LPS treated group, Group C-LPS and Polymyxin B treated group, Group D-LPS and honey treated group, Group E-LPS, Polymyxin B and honey treated group. Control group A showed normal tissue structures with regularly organized neuron cell (NC), pyramidal cell (PC), satellite cell (SC), and blood vessel (BV) in the cortical layer of the cerebrum.But in the case of LPS-treated group B, several nerve fiber degenerations, and spongiosis (Sp) were found. Whereas, neuropil (NP) and glial cells (GC) were normal. In the case of group C, no hemorrhage but mild spongiosis (Sp)wasfound. In the case of group D, most of the cells are normal along with astrocytes (AC) and white matter layer (WML) except mild hemorrhage (MH) and vacuolation. However, in group E, there were no detectable changes in the granular cell layer (GCL), white matter layer (WL) and Purkinje cell (PkC) were also normal.

**Figure 12.**
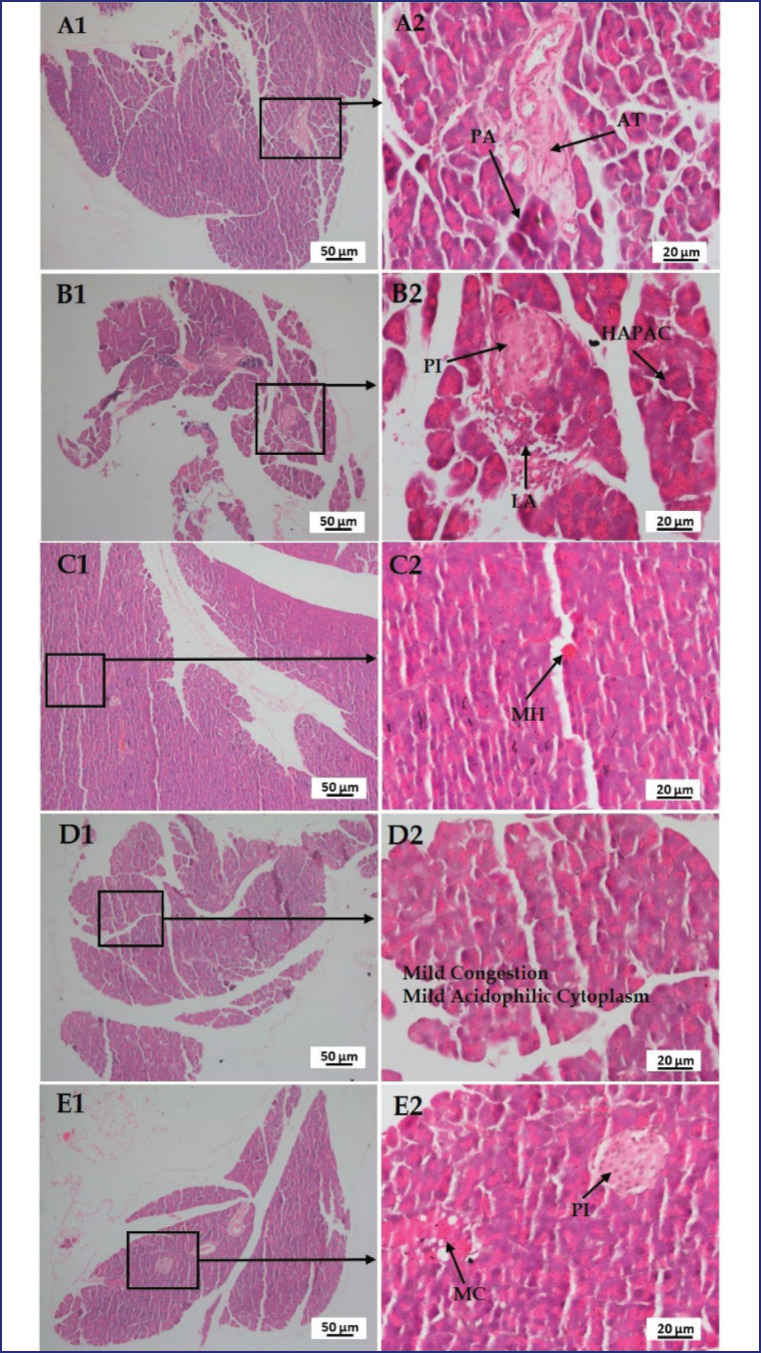
Photomicrograph of histo-structures of pancreas of different treatment groups. Group A-Control, Group B-LPS treated group, Group C-LPS and Polymyxin B treated group, Group D-LPS and honey treated group, Group E-LPS, Polymyxin B and honey treated group. Control group A showed normal tissue structures with regularly organized pancreatic acinar cell (PA) having mild congestion as well as adipose tissue (AT). Whereas, in the case of LPS-treated group B, degenerative changes were found along with lymphoid aggregation (LA) in lymphoid follicles around the pancreatic islets (PI). Hyper acidophilic pancreatic acinar cell cytoplasm (HAPC) was also found. In case of group C, showed minor hemorrhage (MH). In the case of group D, mild congestion (MC) and mild acidophilic cytoplasm was observed. However, in group E, there were no significant changes in pancreatic islets (PI) rather than very less congestion (MC) in pancreatic acini.

A histological assessment of the liver, kidney, lung, brain, and pancreas was carried out to determine the effects of LPS, polymyxin B, and honey on tissue levels of untreated control mice, LPS-treated mice, LPS-plus-polymyxin B-treated mice, LPS plus-honey-treated mice, and LPS-plus-polymyxin B-plus-honey-treated mice. The photomicrographs of the histo-structures of the aforementioned organs of different treated mice at the end of the processing (hematoxylin and eosin-stained) at 10X (A1, B1, C1, D1, and E1) and 40X (A2, B2, C2, D2, and E2) objectives are presented in Figures 8–12.

In Figure 8, a section of the liver of the mice of group A showed normal tissue structures with a regularly shaped hepatic cord and central vein (CV) with normal hepatocytes (H), but in LPS-treated mice, necrotic hepatocytes (NH) along with pyknosis (PyK), cytoplasmic vacuoles (CyV), fragmented nucleus (FN), and inflammatory cells and congestion in the CV. In LPS-plus polymyxin B-treated mice, intact lining endothelial cells (EN denotes endothelial nucleus) around the CV, mild congestion in the CV n CV, and a few numbers of enlarged hepatocytes (EH). Whereas, in LPS-plus honey-treated mice, mild vacuoles (MV) and very minor cellular degeneration were observed. However, in LPS plus polymyxin B plus honey-treated mice, there were no major detectable changes in the hepatic cord or CV.

In Figure 9, a section of the kidney of the mice of group A showed normal tissue structures with regularly formed glomerulus (GL), bowman’s capsular space (BCS), and convoluted renal tubules (RT), but in LPS-treated mice, numerous cellular infiltrations in the GL and hemorrhage in RT. In the case of LPS-plus polymyxin B-treated mice, mild hemorrhage (MH) and relatively less glomerular infiltration. Whereas, in LPS-plus honey-treated mice, there was no hemorrhage and no significant glomerular infiltration. However, in LPS plus polymyxin B plus honey-treated mice, there were no detectable other than a smaller number of cellular infiltrations.

In Figure 10, a section of the lung of the mice in group A showed normal structures of the blood vessel (BV) rather than cuboidal-shaped pneumocytes. Whereas, in the case of LPS-treated mice, distended or ruptured bronchioles (BC) along with peri-bronchiole cellular infiltration and misshaped or emphysematous alveoli were observed. In the case of LPS plus polymyxin B treated mice, MH, peri-bronchial cell infiltration (BC), and normal venioles (BV) with the ratio of RBCs and WBCs. Whereas LPS plus honey-treated mice showed MH in the alveoli. However, in LPS plus polymyxin B plus honey-treated mice, there were no detectable other than a small number of cellular infiltrations around the BC.

In Figure 11, a section of the brain of the mice of group A showed normal tissue structures with regularly structured neuron cells (NC), pyramidal cells (PCs), satellite cells (SCs), and BV in the cortical layer of the cerebrum, but in LPS-treated mice, several nerve fiber degenerations and spongiosis (Sp) were found. Whereas, neuropil and glial cells were normal. In the case of LPS-plus polymyxin B-treated mice, no hemorrhage but mild Sp was found. In the case of LPS plus honey-treated mice, most of the cells are normal, along with astrocytes and the white matter layer (WML), except for MH and vacuolation. However, in LPS plus Polymixin B plus honey-treated mice, there were no detectable changes in the granular cell layer (GCL), WML, or Purkinje cells, which were also normal.

Figure 12 shows a section of the pancreas of mice in group A with normal tissue structures, including regularly ordered pancreatic acinar cells (PA) with mild congestion and adipose tissue (AT). Whereas, in LPS-treated mice, degenerative changes were found, as well as lymphoid aggregation (LA) in the lymphoid follicle around the pancreatic islets (PIs).

Hyper-acidophilic pancreatic acinar cell cytoplasm was also found. Whether, in LPS plus polymyxin B-injected mice, MH and, in the case of LPS plus honey-treated mice, mild congestion and mild acidophilic cytoplasm were observed. However, in LPS plus polymyxin B plus honey-treated mice, there were no significant changes in PI other than very minor congestion (MC) in pancreatic acini.

## Discussion

LPS exposure to Gram-negative bacteria, *E. coli *O55:B5, has sparked interest in assessing health complications, including septic shock. The mortality and morbidity greatly increased in LPS-treated mice due to multiorgan damage and septic shock. Rapid production of reactive oxygen species (ROS), free radicals, cytokines, and chemokines that aggravate inflammation. However, mice treated with polymyxin B and honey and with the combination of these two reduced the effect of LPS, and hence mortality and morbidity rates were also reduced accordingly.

LPS binds three times more to the RBC membrane and activates WBCs, accelerates oxygen-free radical formation, and activates the Toll-like receptor (TLR-4), causing complement-mediated hemolysis and reducing erythrocytes, potentially inhibiting hematopoiesis [16]. LPS binds more to RBC membranes due to biochemical alterations caused by free radicals and cytokines. That decreases membrane elasticity and deformability and may lead to a loss of normal membrane flexibility [16]. Alongside RBCs, the Hb concentration and PCV are also reduced. Whereas PCV depends on the erythrocyte mass, mean corpuscular volume, and plasmatic volume. In contrast, oxidation of Hb may contribute to the reduction of RBC deformability in septicemia due to the treatment with polymyxin B sulfate and honey separately and treatment with both polymyxin B and honey. That is why there might be improvements in the values of RBC, Hb concentration, and PCV compared to the LPS-treated mice. Previous researchers observed a significant decrease in RBC count, Hb concentration, and PCV, indicating an anemic condition, according to the results. The significant reduction in lymphocyte percentage in LPS-treated mice is due to suppressor B cell activation. Recovering the LPS effect with polymyxin B and honey increased the percentage by inhibiting B cells, which aligns with other studies. The rise of neutrophils and monocytes due to LPS exposure may be associated with an acute inflammatory response, triggering the release of cytokines and inflammatory cells like neutrophils, monocytes, and macrophages. LPS is a potent activator and numerator of these cells. Whereas neutrophils and monocytes are crucial in endotoxin-induced shock and host defense, The study investigates whether treatment with polymyxin B, honey, or combined treatments neutralizes bacterial LPS in mice. Therefore, the values of neutrophils and monocytes decreased significantly compared to only LPS-treated mice.

The increased AST, ALT, and ALP values due to inflammatory cytokines in mice treated with LPS indicate liver injury, which aligns with previous studies indicating LPS-induced inflammatory response, hepatocyte necrosis, and inflammatory cell infiltration [10]. In contrast, the reduction in values on exposure to polymyxin B and honey may be because of its antioxidant and antibacterial effects, which prevent the release of inflammatory cytokines. Usually, TNFα, cytokines, and chemokines are synthesized within the tubular epithelium of the kidney, causing inflammation and renal dysfunction. LPS synthesizes nitric oxide and PGE2, inflammatory cytokines like TNFα. Studies have indicated that treatment with antioxidant and anti-inflammatory agents is beneficial for LPS-induced renal injury. The study investigated the mechanisms behind polymyxin B and honey’s ability to prevent acute kidney injury in LPS-injected mice. A prior study found that cytokine production was suppressed by reducing inflammatory signaling and stress-activated mitogen-activated protein (MAP) kinases, enhancing endotoxemia. It is confirmed that treatment with polymyxin B and honey significantly suppressed the damage to the renal tissues compared to the LPS-treated mice. The current investigation found that LPS dramatically reduced serum glucose levels by inhibiting both glucose synthesis and glucose consumption. Although multiple tracer studies have indicated that acute LPS exposure reduces glucose synthesis, there is conflicting data on how LPS affects glucose uptake. In one investigation, the effect of LPS on glucose synthesis and utilization was assessed, and glucose uptake was reduced during LPS-induced hypoglycemia but enhanced when euglycemia was restored by glucose infusion. On the other hand, serum insulin levels were significantly increased in LPS-treated mice in response to non-glucose substrates such as propionate, butyrate, or acetic acid, which may increase during LPS-induced inflammation by stimulating beta cells of the islets of Langerhans. These findings align with other studies [17]. Insulin levels were dependently elevated in L150 ewes, but polymyxin B and honey restored them by reducing endotoxemia effects in LPS-treated mice.

The histo-texture of the liver can be compared to previous studies that stated that LPS causes monocellular infiltrations, some enlarged hepatocytes [18], finely granular cytoplasm, the collapse of sinusoids between hepatocytes, some hepatocytes with karyolytic changes, pyknosis, cytoplasmic vacuoles, and a dilated congested CV [5]. LPS-induced acute liver injury occurs due to inflammatory mediators such as TNF-α, IL-1β, and ROS, affecting mitochondrial function and hepatocyte apoptosis, affecting Kupffer cells and hepatocytes. However, studies have shown that treatment with antioxidant and anti-inflammatory drugs such as honey is effective in reducing LPS-induced hepatic damage. However, excessive honey consumption may interfere with the liver’s natural histological alterations [19]. Mahalaway [20] observed that mice treated with LPS plus propolis (honey) had intact hepatic architecture, including more or less typical hepatocytes, CVs, and blood sinusoids. The study reveals that polymyxin B may alter hepatic abnormalities, but when paired with honey, it protects against LPS-induced liver damage. The findings are aligned with those of other scientists who stated that LPS causes vacuolar degeneration and necrosis in epithelial cells in RT, damaged brush borders, and the presence of cellular infiltration in the GL and hemorrhage in the RT [21]. The histo-texture was altered by TNF-α release, an inflammatory cytokine responsible for cell signaling events, leading to necrosis or apoptosis. Serum TNF-α levels can peak as early as 0.5–2 h following LPS injection, according to multiple studies [21].

It was also noticed that the same incident occurred in this study. Although polymyxin B sulfate has submissive effects on LPS, slight cellular infiltrations in renal cells are believed to be due to its nephrotoxic effect. Whereas honey reduces tissue injury by reducing inflammatory cytokines and chemokines, and its antioxidant and antimicrobial properties preserve kidney histo-structures compared to LPS-treated mice. However, the histological features of the lung can be compared to prior research that claimed that LPS produces large inflammatory cell infiltration, alveolar congestion, thickening of the alveolar walls, and hemorrhage [22]. The activation of ROS, cytokines, TNF-alpha, MAP kinases, and other transcription factors might be the major factors in altering the tissue structure. Whereas polymyxin B reduces multifocal lesions, variable degrees of congestion, and inflammatory cells by reducing pro-inflammatory cytokines. However, the honey or polymyxin B- and honey-treated mice showed a protective nature by diminishing ROS, cytokines, and TNF-alpha. In the case of the histo-structures of the brain, the findings are consistent with those of other investigators who stated that LPS causes nerve degeneration and darker neuron cells, irregular cells with shrunken cytoplasm, Sp, and hemorrhage. ROS causes axonal degeneration and circadian disruption [23].

Pro-inflammatory cytokines attracted macrophages that bound to TLRs. Ultimately, damage to nervous tissue occurred. Whereas pyramidal cell loss and remarkable neuronal abnormalities were prevented in the honey-treated mice due to less ROS and cytokine production [24]. It also noticed the protective action of polymyxin B and honey against LPS and found positive outcomes. The histo-texture of the pancreas can be compared to previous studies that stated that LPS causes interstitial edema, a few cytoplastic vacuoles, and PA cell necrosis [17]. But LA in PI lesions may be caused by co-infection with bacteria or unknown factors, aggravated by inflammatory cytokines and chemokines. However, Ramli et al. [25] reviewed that honey reduces cytoplasmic degenerative changes in PI of Langerhans. Mice treated with polymyxin B or honey minimized LPS effects, but combined treatments were more protective by reducing ROS, free radicals, cytokines, and chemokines.

## Conclusion

The research findings indicate that LPS exposure is a risk factor for hemato-biochemistry. Polymyxin B and honey may influence the treatment of anemia. It affects different organs’ histological structures. As a result, avoiding exposure to LPS is critical for animal health. Polymyxin B and honey consumption may play an important role in reducing the negative effects of LPS. The original insights from this study are predicted to open a new door in the research area and may serve as a research track for filling information gaps about LPS, polymyxin B, and honey, particularly in developing countries. However, more research is needed to reduce the health risks associated with LPS exposure and to determine the precise mechanism of action of polymyxin B and honey against it.

## List of Abbreviations

ALP, Alkaline Phosphatase; ALT, Alanine Transaminase; AST, Aspartate Transaminase; Bwt, Body Weight; Cell; SCr, Serum Creatinine; DLC, Differential Leukocyte Count; Hb, Hemoglobin; LPS, lipopolysaccharide; PCV, Packed Cell Volume; ROS, Reactive Oxygen Species; RBC, Red Blood Cell; TEC, Total Erythrocyte Count; TLC, Total Leukocyte Count; WBC, White Blood Cell.
